# Limonin Isolated From Pomelo Seed Antagonizes Aβ25-35-Mediated Neuron Injury via PI3K/AKT Signaling Pathway by Regulating Cell Apoptosis

**DOI:** 10.3389/fnut.2022.879028

**Published:** 2022-05-12

**Authors:** Yuanxin Qiu, Jingxian Yang, Lukai Ma, Mingyue Song, Guo Liu

**Affiliations:** ^1^School of Food Science and Engineering, Zhongkai University of Agricultural Engineering, Guangzhou, China; ^2^Guangdong Meizhou Vocational and Technical College, Meizhou, China; ^3^Guangdong Provincial Key Laboratory of Lingnan Specialty Food Science and Technology, Guangzhou, China; ^4^Guangdong Provincial Key Laboratory of Nutraceuticals and Functional Foods, College of Food Science, South China Agricultural University, Guangzhou, China

**Keywords:** pomelo seed, limonin, PC12 cells, neuroprotective activity, PI3K/AKT

## Abstract

Pomelo seed as a by-product from pomelo consumption is rich in bioactive compounds, however, a huge volume of pomelo seed was disposed as wastes, the comprehensive utilization of pomelo seed could not only generate valued-added products/ingredients, but also decrease the environmental pollution. In this study, the main active substance limonin in pomelo seed was considered as a high-value bioactive compound. The purification of limonin from pomelo seed was investigated, and the neuroprotective and mechanism were characterized. The UPLC-MS/MS results indicated that 29 compounds in pomelo seed were identified, including 14 flavonoids, 3 limonids, 9 phenols and 3 coumarins. Moreover, high purity of limonin was obtained by crystallization and preparative-HPLC. Furthermore, limonin pretreatment can antagonize the cell damage mediated by Aβ_25−35_ in a concentration-dependent relationship. The regulation of Bax/Bcl-2, expression of caspase-3 protein and the activation of PI3K/Akt signaling pathway were observed in the cells pretreated with limonin. Treatment of PC12 cells with PI3K inhibitor LY294002 weakened the protective effect of limonin. These results indicated that limonin prevented Aβ_25−35_-induced neurotoxicity by activating PI3K/Akt, and further inhibiting caspase-3 and up-regulating Bcl-2. This study enables comprehensive utilization of pomelo seed as by-product and offers a theoretical principle for a waste-to-wealth solution, such as potential health benefits of food ingredient and drug.

## Introduction

Pomelo [*Citrus grandis (L.)* Osbeck], known as shaddock and pummel, is one of the most important commercially fruit crops. Pomelo belongs to the citrus genus in the Rutaceae family, which is widely cultivated in Asia and some other countries around the world (1–3). Its cultivation and consumption can date back to more than 4,000 years (4). In 2018, a total of 9.4 million metric tons of pomelo was reportedly produced in global according to the Food and Agricultural Organization of the United Nations database (5). Pomelo seeds, as the main by-product of pomelo, account for ~10.3% of the entire pomelo (6). In China, pomelo seeds are used in the treatment of hernias, cold lungs, coughs, yellowing, and breast hyperplasia due to its phytochemicals (6, 7). However, only small amounts of pomelo seeds are currently used as raw materials for traditional Chinese medicine due to their bitter taste. As a result, most of these pomelo seeds are discarded. Thus, the overall health benefits and economic value of pomelo seeds have not been fully appreciated. Most pomelo seeds go into landfill, which caused a serious environmental problem. Limonoids, flavonoids and polyphenols are the major chemical components in pomelo seeds. Previous studies have shown that these compounds have a variety of biological activities, such as anti-oxidation and anti-inflammatory effects, scavenging free radicals, inhibiting cancer cell growth and enhancing the activity of nerve growth factors (8–10). Therefore, pomelo seed extracts have great potential as a dietary nutritional supplement.

Limonin, as a triterpenoid compound, is the most prevalent member of limonoids in the citrus genus. It shows a broad-spectrum of biological activity, especially neuroprotective effects (11). Some previous study reported that limonin exhibited remarkable neuroprotective activity against glutamate-induced neurotoxicity *in vivo* (12) and *in vitro* (13). Triterpenoid derivatives are also considered to be important active substances with anti-β amyloid deposition (14). A recent study revealed that limonin could improve neurodegenerative lesions in rats by improving memory and the ability to learn (15).

The average age of the world population has been increasing in the last few decades, which also increases the possibility of the elderly to suffer from dementia. Alzheimer's disease (AD) accounts for 50–70% of all dementia (16). Currently, the most acceptable hypotheses include the cholinergic hypothesis, the β-amyloid hypothesis, and the Tau protein hypothesis (17). Among them, amyloid beta (Aβ) is the main component of senile plaques which are composed of 36–43 amino acid residues (18). A large amount of medical evidence shows that Aβ plays an indispensable role in the pathogenesis of AD. Aβ oligomers, such as Aβ_25−35_ fragments, can lead to neuronal dysfunction (19–21) by causing mitochondrial dysfunction, imbalance of Ca^2+^, and the occurrence of neuronal oxidative stress damage. Additionally, a potential toxic substance can cause neuronal dysfunction and neuron loss by severely damaging synaptic structure and function (22). Aβ_25−35_ fragments have shown neurotoxicity in cell culture (23). Therefore, Aβ_25−35_ has been widely used to establish *in vitro* AD experimental models. Although drugs such as donepezil can be used for the treatment of AD, these drugs usually have serious side effects such as drowsiness, restlessness, arrhythmia, and epilepsy. This drives people to pursue natural nutritional supplements with low side effects. To date, there are only a few reports on the mechanism of limonin and its effect on nerve cells. Further studies can help understand this mechanism and lead to better nutrition intervention for neurodegenerative diseases.

In the present study, the main bioactive compounds in pomelo seed were identified using the UPLC-MSMS system. Then, the neuroprotective effect of limonin isolated from pomelo seed was investigated using an apoptosis model of PC12 cells induced by Aβ_25−35_. This study enables comprehensive utilization of the waste pomelo seed resources and proves the neuroprotective effect of limonin in the current scientific literature.

## Materials and Methods

### Materials and Chemicals

The pomelo seeds were obtained from Meizhou, Guangdong province, China. Chromatographic grade acetonitrile was obtained from Merck (Merck KGaA, Darmstadt, Germany), trifluoroacetic acid, ethanol, formic acid, ethyl acetate, dichloromethane and isopropanol were obtained from Guangzhou Chemical Reagent Factory (Guangzhou, China). RPMI-1640 cell medium, fetal bovine serum and penicillin were all purchased from Sigma-Aldrich (St. Louis, MO, USA). Fluo-3/AM and LY294002 (the inhibitor of PI3K) were acquired from Shanghai Beyotime Biotechnology (S1056, Beyotime, China) and Selleck Chemicals (S1105, Selleck, USA), respectively.

### Extraction and Identification of Bioactive Compounds

Subcritical equipment (Henan Subcritical Biotechnology, China) was used to remove grease of pomelo seeds with n- butane. The conditions were as follows: material liquid ratio 1: 4 (g/mL), extraction time 50 min, extraction temperature 50°C, and extraction 2 times.

The defatted pomelo seed (dried, 1.2 kg) was extracted three times with 70% ethanol at 50°C for 4 h. The ethanol extracts were analyzed by the UPLC-MSMS system (Thermo Fisher Scientific, USA). Chromatography was carried out on Zorbax Eclipse C18 (1.8 μm^*^2.1^*^100 nm, Agilent technologies, USA) at 30°C. The mobile phase contained 0.1% formic acid-water (A) and acetonitrile (B). The flow rate was 0.3 mL/min and injection volume was 2 μL. The gradient conditions were set as follows: 0–5% (B) in 0 to 2 min, 2–30% (B) in 2–6 min, 30% (B) in 6–7 min, 30–78% (B) in 7–12 min, 78% (B) in 12–14 min, 78–95% (B) in 14–17 min, 95% (B) in 17–20 min, 95–5% (B) in 20–21 min, and 5% (B) in 21–25 min.

The mass spectra were performed in positive electrospray ionization (ESI) modes, and the optimal conditions were set as follows: heater temperature 325°C; sheath gas flow rate 45 arb; auxiliary gas flow rate 15 arb; purge gas flow rate 1 arb; electrospray voltage 3.5 KV; capillary temperature 330°C; S-Lens RF Level 55%. Scanning mode was primary full scanning (Full Scan, m/z 100–1,500) and data-dependent secondary mass spectrometry scanning (dd-MS2, TopN = 10). Resolution was 120,000 (primary mass spectrometry) and 60,000 (secondary mass spectrometry). Collision mode was high-energy collision dissociation (HCD).

### Purification of Limonin

The ethanol extracts (118 g) was obtained after drying under vacuum condition at 50°C in a water bath, and then further extracted with ethyl acetate. These extracts were once again concentrated in a vacuum evaporator. The extracts was resolved using dichloromethane and isopropanol (1:2 v/v), then recrystallized at 4°C for 8 h. The recrystallization operation was performed three times until pure white crystals were obtained. The obtained filtrates were concentrated and recrystallized to obtain the mother liquor. The preparative-HPLC (Yilite,China) was used later for separation and the analytical-HPLC (1100, Agilent technologies, USA) for analysis to give compound (101 mg). The preparative-HPLC conditions contained water (A) and acetonitrile (B). The flow rate was 3 mL/min and injection volume was 500 μL. The gradient conditions were set as follows: 0–10% (B) in 0 to 5 min, 10–45% (B) in 5–10 min, 45% (B) in 10–30 min. The purification of limonin was calculated by area normalization method.

### Test of Neuroprotective Effect on PC12 Cells

#### Cell Culture and Treatment

Aβ_25−35_ (HY-P0128, MCE, China) stock solution (180 μmol/L) was prepared as follows: Aβ_25−35_ was dissolved in distilled water, and aliquots were stored at −20°C and incubated at 37°C for 7 days before usage. Highly differentiated rat pheochromocytoma PC12 cells (iCell-r024, iCell Bioscience Inc, China) were cultured at 37°C with 5% CO_2_ in RPMI-1640 medium with 1% penicillin and 10% fetal bovine serum.

Cell Counting Kit-8 (CCK-8) was applied to determine the final dosage of Aβ_25−35_ and limonin for cell treatment. Results showed that treating cells for 48 h with 20-μM of Aβ_25−35_ significantly inhibited PC12 cell growth. Therefore, 20 μM Aβ_25−35_ was chosen as the cell injury model concentration.

Cells were divided into the following groups: control, Aβ_25−35_, Aβ_25−35_+12.5 μg/mL limonin, Aβ_25−35_+25 μg/mL limonin, Aβ_25−35_+50 μg/mL limonin, Aβ_25−35_+LY294002, and Aβ_25−35_+LY294002+50 μg/mL limonin. In the control group, only cell culture medium was added. In Aβ_25−35_ group, the cells were treated with 20 μM Aβ_25−35_ for 48 h. In the sample groups, the cells were pre-treated with various concentrations of limonin for 1 h. After that, 20 μM Aβ_25−35_ was added and cultured for 48 h. In Aβ_25−35_+LY294002 and Aβ_25−35_+LY294002+50 μg/mL limonin groups, the cells were treated with LY294002 for 1 h before the addition of Aβ_25−35_ and limonin, and then incubated for 48 h.

#### Cell Viability Assay

CCK-8 assay was used to determine cell viability. The PC12 cells were seeded into 96-well plates at a density of 1 × 10^4^ cells/well and then incubated with Aβ_25−35_, limonin and LY294002 for 48 h. After incubation, 20 μL of CCK-8 reagent was added and incubated for 2 h. Next, the upper medium was removed and absorbance was measured at 450 nm using a Microplate Reader (DR-200Bs, Diatek, China).

#### Lactic Dehydrogenase (LDH) Cytotoxicity Assay

PC12 cells were inoculated into a 6-well plate and cultured for 24 h, and then processed in different groups and incubated for a certain amount of time. After the incubation, cells were collected and washed twice with phosphate buffered saline (PBS). The LDH ratio of each group was measured using a LDH kit (A020-1-2, NanJing JianCheng Bioengineering Institute, China).

#### Ca^2+^ Concentration Assay

The intracellular Ca^2+^ concentration assay was used as previously described method with some modifications (24). Briefly, at the end of incubation, 5 μmol/L Fluo-3/AM working solution (prepared in DMSO) was added to each well and incubation continued in the dark; then the Fluo-3/AM working solution was removed, and the cells were washed with Hank's balanced salt solution without Ca^2+^ buffer. This step was repeated until Fluo-3/AM was fully converted, and the fluorescence intensity was detected by a flow cytometer (AriaIII, BD, USA).

#### Reactive Oxygen Species (ROS) Activity Assay

Intracellular ROS levels were detected using a reactive oxygen species detection kit (S0033, Bryotime, China). Briefly, after different treatments, cells were collected and 10 μM 2,7-Dichlorodihydrofluorescein diacetate (DCFH-DA) working solution diluted with serum-free medium was added, mixed well and incubated at 37°C in the dark for 30 min. Then, the cells were washed three times with PBS and analyzed by flow cytometry.

#### Measurement of Mitochondrial Membrane Potential (MMP)

Mitochondrial membrane potential was detected using a MMP detection Kit (C2006, Beyotime, China). Briefly, after different treatments, cells were harvested and 1 mL JC-1 solution was added and incubated at 37°C for 20 min. After the incubation, cells were washed three times with PBS, resuspended, and analyzed by flow cytometry.

#### Apoptosis Assay

Apoptosis was measured using Annexin V-FITC Apoptosis Detection Kit (AO2001-02P-G, Sungene Biotech, China). Briefly, after different treatments, cells were harvested, binding buffer (300 μL) diluted with PBS was added, and then 5 μL of PI and 5 μL Annexin V-FITC were added and incubated in the dark for 15 min, followed by measurement using flow cytometry: FITC excitation 494 nm, emission 520 nm; PI excitation 493 nm, emission 636 nm.

#### Western Blot Analysis

Western blotting assay was used to analyze changes of cell signaling. Briefly, at the end of incubation, the cells were harvested and Protein Extraction Kit (AS1011, ASPEN, China) was used to extract total cell proteins by following the manufacturer's instructions. BCA Protein Concentration Determination Kit (AS1086, ASPEN, China) was used to determine the total protein concentration. Protein samples were then separated on 12% SDS-PAGE and transferred to PVDF membranes (IPVH00010, Millipore, USA). β-actin served as an internal control. After blocking with 5% skimmed milk at room temperature for 1 h, the blocking solution was removed, and the membranes were washed with TBST buffer. PVDF membranes were then incubated with primary antibodies overnight at 4°C, and then washed three times with TBST buffer, and further incubated with secondary antibodies. The freshly prepared ECL mixed solution (A : B = 1 : 1) was used for membrane exposure in a dark room. The optical densities of the target bands were analyzed using the commercial AlphaEaseFC software.

### Statistical Analysis

All the data were analyzed using SPSS 25.0 software and are expressed as mean ± standard (SD). The differences between treatments were assessed by one-way ANOVA. *P-*value < 0.05 was considered statistically significant.

## Results

### Ethanol Extract Identification Using UPLC-MSMS

**Figure 1:**
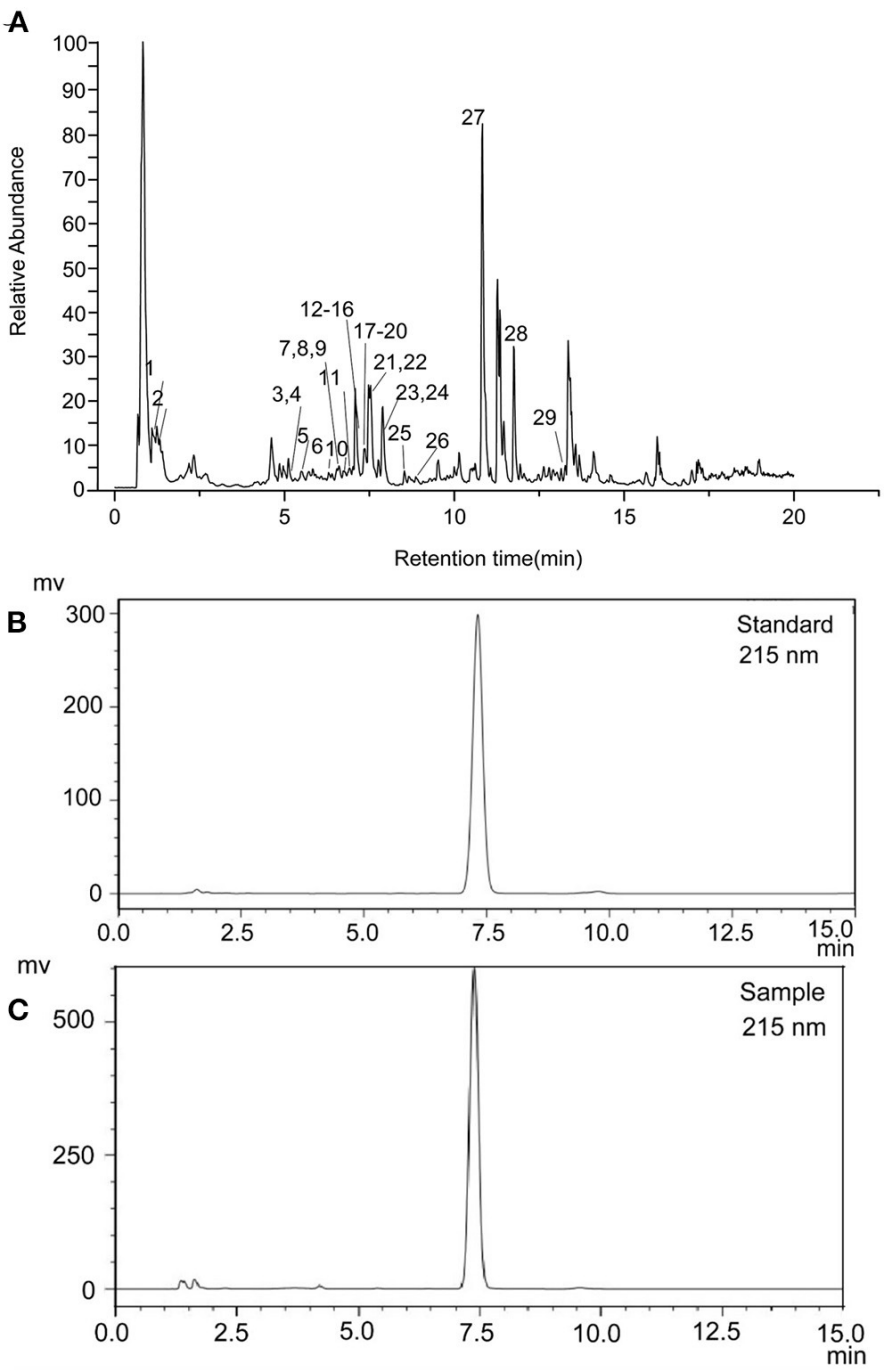
Total ion current chromatograms of pomelo seed extract detected in positive ion mode **(A)**. HPLC chromatogram of limonin standard **(B)** and sample **(C)**.

The ethanol extract from the pomelo seed was identified using UPLC-MSMS. The TIC chromatograms of the various compounds of ethanol extract from the pomelo seed was shown in Figure 1. These results indicated that a total of 29 compounds had been identified by LC-ESI/MS and their retention times (t_R_), MS, and MS/MS fragmentation ions are shown in Table 1, including 14 flavonoids, 3 limonids, 9 phenols, 3 coumarin and its derivatives. The two peaks with the highest response values were selected for analysis. Results showed that the compound (t_R_=0.85) exhibited quasi-molecular ion peaks [M+H]^+^ at m/z 144.1019 in the MS spectra, using Agilent qualitative analysis software to collect the molecular information of the component ions. The molecular formula of this component was C_7_H_13_NO_2_. Meanwhile, different levels of collision energy were used to obtain appropriate MS/MS information, and the characteristic fragment ions were at m/z 84.08133 (C_5_H_9_N) and m/z 98.09681 (C_6_H_11_N). Through the secondary mass spectrum, using the Thermo mzCloud online database, the compound was preliminarily identified as DL-Stachydrine. Another substance was identified in the same way; the molecular formula was C_26_H_35_O_8_, ion m/z 471.2009 [M+H]^+^, and the characteristic fragment ions were at m/z 425.1951 (C_25_H_33_O_6_) and m/z 95.0496 (C_5_H_7_O_2_); this compound was tentatively identified as limonin. The TIC ion chromatogram showed that the response value of limonin was very high.

**Table 1:** The characteristic components and their recognition results filtered by frequency in principal component analysis.

**Peak** **number**	**Proposed compounds**	**Retention time (min)**	**Molecular formula**	**Molecular mass**	**Mass-to charge ratio**	**Deviation** **(ppm)**	**MS/MS fragment ions relative abundance (100%)**
1	Phloroglucinol	1.237	C_6_ H_6_ O_3_	126.03176	127.03905[M+H]^+^	0	109.02875, 99.04456, 81.03412
2	2-Hydroxycinnamic acid	1.267	C_9_ H_8_ O_3_	164.04742	165.05470[M+H]^+^	0	119.04931
3	4-Methylumbelliferyl-α-D-glucopyranoside	5.477	C_16_ H_18_ O_8_	338.09993	339.10715[M+H]^+^	0	147.04401
4	Esculetin	5.481	C_9_ H_6_ O_4_	178.02658	179.03383[M+H]^+^	0	133.00283
5	Luteolin-3',7-Diglucoside	5.546	C_27_ H_30_ O_16_	610.15343	611.16071[M+H]^+^	0	450.11090,288.05798
6	5,7-Dihydroxy-4-methylcoumarin	6.378	C_10_ H_8_ O_4_	192.04235	193.04962[M+H]^+^	0	178.02595,165.05472,149.0232
7	Isoferulic acid	6.556	C_10_ H_10_ O_4_	194.05793	195.06532[M+H]^+^	0	177.05458,163.03891,149.09610,145.00284
8	Vanillin	6.607	C_8_ H_8_ O_3_	152.04735	153.05463[M+H]^+^	0	125.05981
9	Eriodictyol	6.633	C_15_ H_12_ O_6_	288.06308	289.07031[M+H]^+^	−1	153.01820,123.04420,187.03896,179.03381,265.04933,243.0975
10	Quercetin	6.737	C_15_ H_10_ O_7_	302.04244	303.4971[M+H]^+^	0	274.0467,229.0941,165.01823,153.0181,137.02327
11	Ferulic acid	6.947	C_10_ H_10_ O_4_	194.05792	195.06522[M+H]^+^	0	177.05455,163.03893,149.05969,145.02837,117.03369
12	Keracyanin	7.028	C_27_ H_30_ O_15_	594.1583	595.16553[M+H]^+^	0	576.8625,552.69696,450.11115,449.10754,129.05479
13	Rhoifolin	7.041	C_27_ H_30_ O_14_	578.16333	579.17072[M+H]^+^	0	434.11575,271.05991,151.10870
14	Prunin	7.091	C_21_ H_22_ O_10_	434.12077	435.12802[M+H]^+^	−1	273.0753,153.01814
15	Naringeninchalcone	7.092	C_15_ H_12_ O_5_	272.06802	273.0752[M+H]^+^	−1	153.01822,107.04946
16	Naringin	7.092	C_27_ H_32_ O_14_	580.17873	581.18579[M+H]^+^	0	273.07538
17	Kaempferol	7.103	C_15_ H_10_ O_6_	286.04735	287.05466[M+H]^+^	−1	241.04906,213.05424,165.01826,153.01825,121.02847
18	Kaempferol-7-O-glucoside	7.107	C_21_ H_20_ O_11_	448.10019	449.10751[M+H]^+^	0	287.0548
19	Isorhamnetin	7.188	C_16_ H_12_ O_7_	316.05789	317.06522[M+H]^+^	−1	302.0418,285.03909,274.04694,153.01814
20	Hesperetin	7.195	C_16_ H_14_ O_6_	302.07875	303.08599[M+H]^+^	0	285.07520,177.05446,153.0180,
21	Sinapinic acid	7.463	C_11_ H_12_ O_5_	224.06842	225.07561[M+H]+	0	207.0653,192.04147,147.04395
22	Naringenin	7.533	C_15_ H_12_ O_5_	272.06803	271.06122[M+H]^+^	−1	255.06500,179.03391,153.001823,147.044005,119.0494
23	Sinapyl aldehyde	7.636	C_11_ H_12_ O_4_	208.07347	209.08075 [M+H]^+^	0	177.05412, 191.07382, 121.06431
24	4-Coumaric acid	7.776	C_9_ H_8_ O_3_	164.04728	165.05455[M+H]^+^	0	147.0439,119.04929
25	Pinocembrin	8.616	C_15_ H_12_ O_4_	256.07329	257.08054[M+H]^+^	−1	153.01816,131.04918
26	Betaxolol	8.861	C_18_ H_29_ N O_3_	307.21442	308.22171[M+H]^+^	−1	116.1071, 72.0806, 98.0964
27	Limonin	10.568	C_26_ H_30_ O_8_	470.19365	471.20099[M+H]^+^	0	425.19510,95.04965
28	Obacunone	11.759	C_26_ H_30_ O_7_	454.19857	455.20581[M+H]^+^	−1	161.05966, 409.20065
29	Nootkatone	13.036	C_15_ H_22_ O	218.16692	219.17418[M+H]^+^	0	163.11166

### Purification of Limonin

Limonin was principally recovered by isolation and purification. In this study, limonin was obtained with a purity of 99.13% after recrystallization and pre-HPLC in Figure 1C. The limonin was identified by HPLC and comparison with authentic standards retention time (t_R_) in Figure 1B.

### Morphological Changes of PC12 Cells

**Figure 2:**
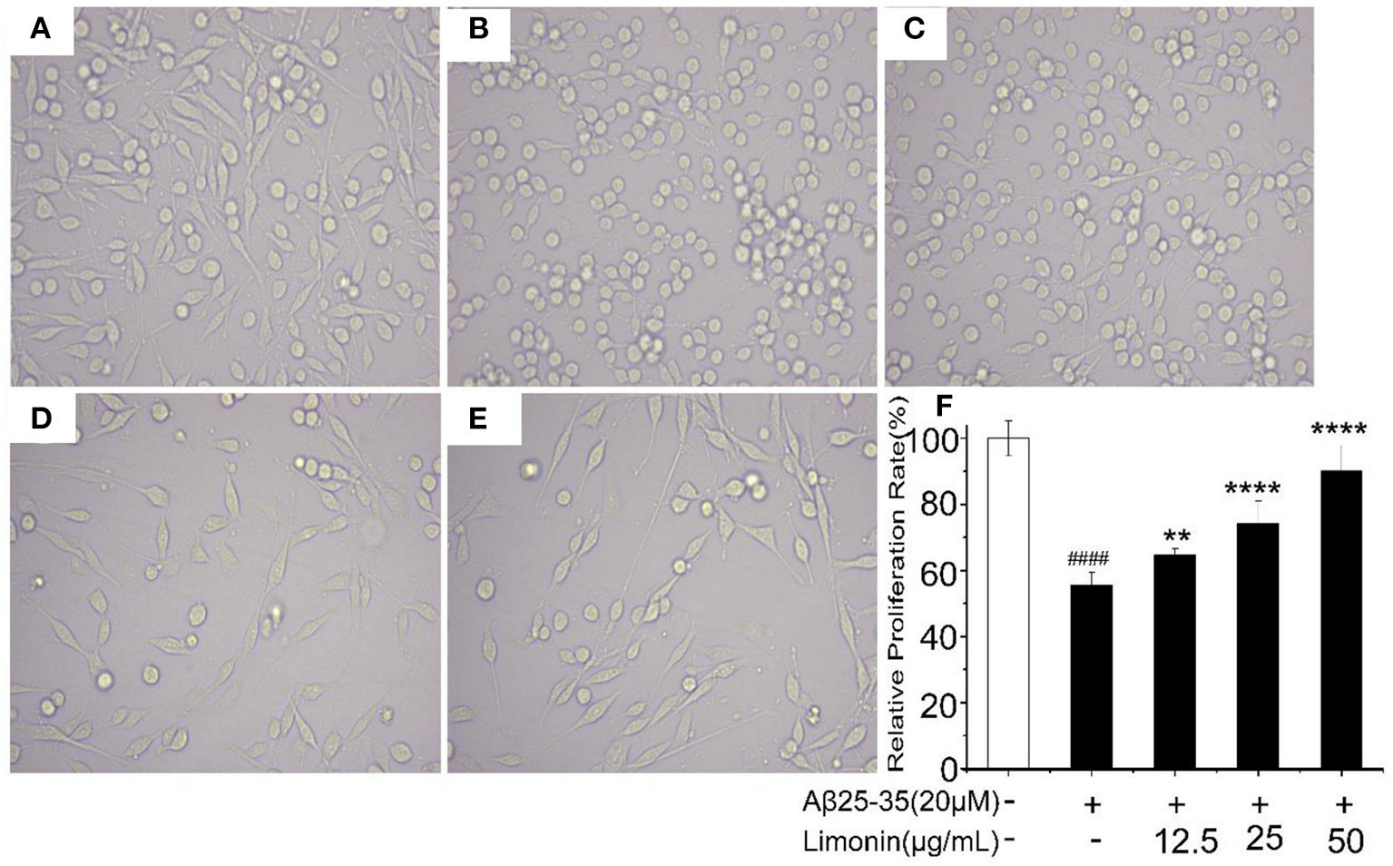
Morphological changes of PC12 cells with different treatments. **(A)** Control group; **(B)** Aβ_25−35_ group; **(C)** Aβ_25−35_ +12.5 μg/mL limonin group; **(D)** Aβ_25−35_ +25 μg/mL limonin group; **(E)** Aβ_25−35_ +50 μg/mL limonin group; **(F)** Cell viability determination by CCK-8 assay. ####*p* < 0.0001 compared with the control group; **p < 0.01 and ****p < 0.0001 compared with the Aβ_25−35_ group.

The morphological changes of PC12 cells are shown in Figure 2. The cells in the control group adhered well and grew like a spindle with a clear cell outline. After treatment with Aβ_25−35_, the shape of the cells changed significantly, most of the cell protrusions were broken and the edges blurred. While pre-treatment of limonin significantly alleviated the injuries caused by Aβ_25−35_ and maintained normal cell morphology.

### LDH Release

**Figure 3:**
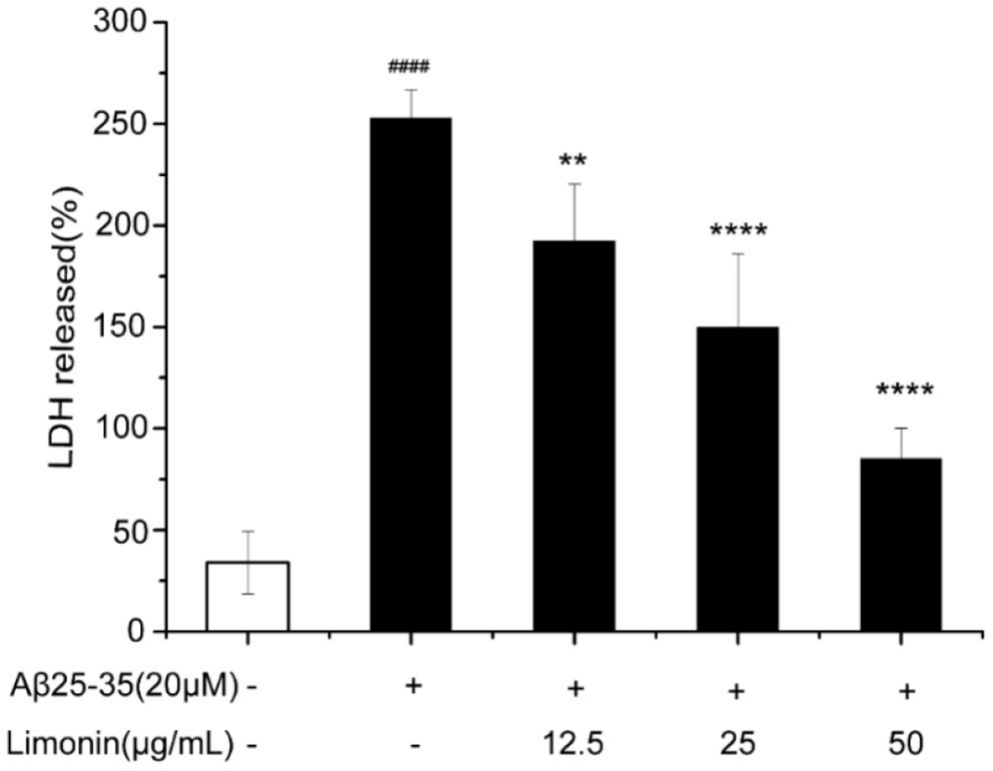
Lactic dehydrogenase (LDH) activity was measured using an LDH assay kit. ####*p* < 0.0001 compared with the control group; **p < 0.01 and ****p < 0.0001 compared with the Aβ_25−35_ group.

As shown in Figure 3, when compared to the control group cells, treatment of cells with 20 μM Aβ_25−35_ for 48 h resulted in a significant inhibition of cell viability and a significant increase in the release of LDH (*P* < 0.0001). Pre-treatment of cells with limonin reduced Aβ_25−35_-mediated cell injury and the release of LDH (*p* < 0.0001). In the Aβ_25−35_ + limonin group at doses of 12.5, 25, 50 μg/mL, the release of LDH declined from 192.59% to 85.19%. The results showed that as the concentration of limonin increased so did the protective effect on PC12 cells. The highest protection was observed when 50 μg/mL of limonin was utilized (*p* < 0.0001).

### [Ca^2+^]I Release

**Figure 4:**
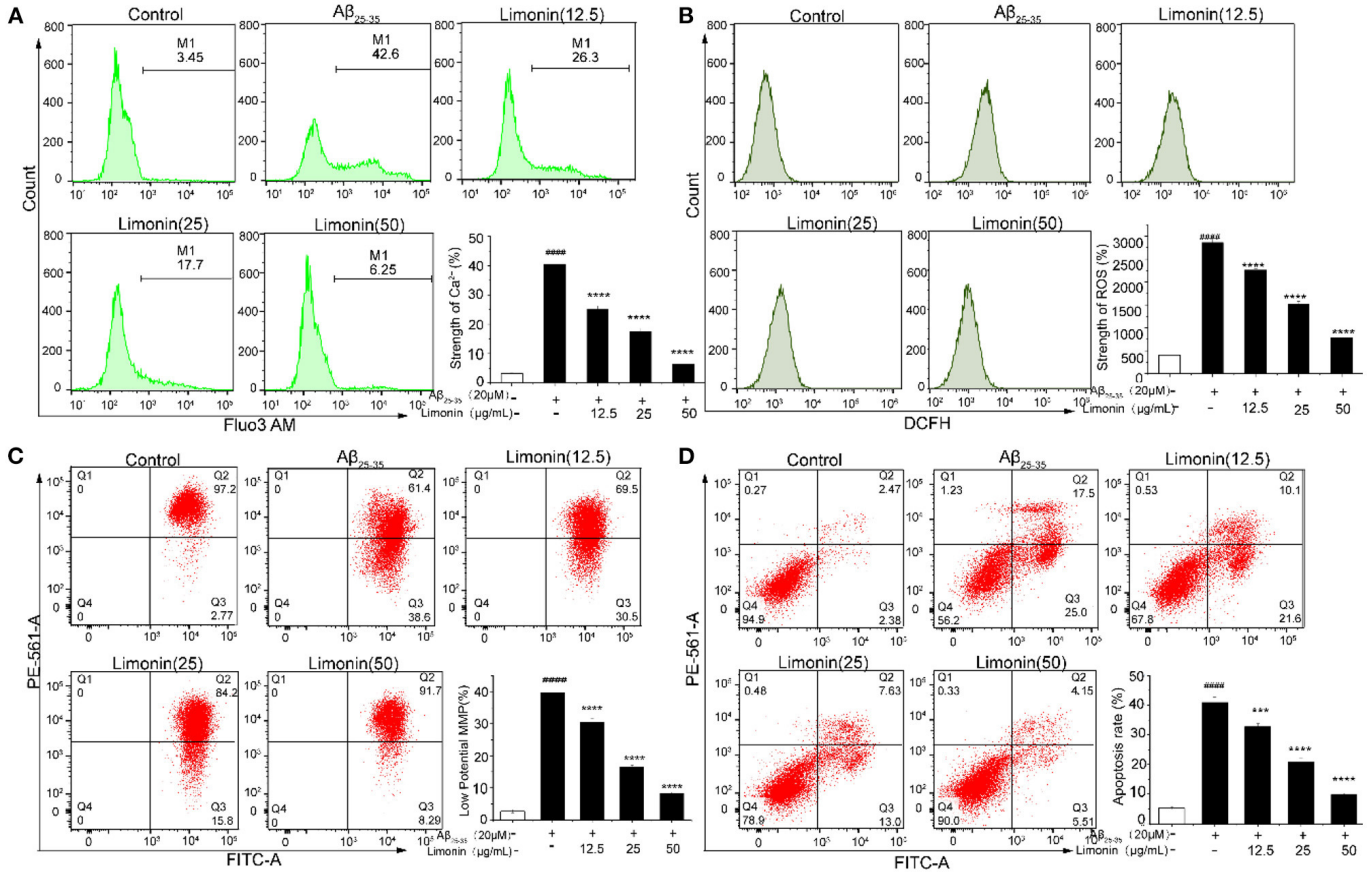
The effect of limonin on [Ca^2+^]i, ROS, MMP and apoptosis of PC12 cells induced by Aβ_25−35_ treatment, analyzed with flow cytometer. **(A)** [Ca^2+^]i release; **(B)** reactive oxygen species (ROS) levels; **(C)** mitochondrial membrane potential (MMP); **(D)** apoptosis. ####*p* < 0.0001 compared with the control group; ***p < 0.001 and ****p < 0.0001 compared with the Aβ_25−35_ group.

As shown in Figure 4A, when compared to the control group cells, for the PC12 cells that were treated with Aβ_25−35_, the content of [Ca^2+^]i increased significantly from 3.26 to 40.37%, indicating that the Ca^2+^ in the cells were overloaded. The pre-treatment of limonin induced a statistically significant decrease (*p* < 0.0001*)* of intracellular Ca^2+^ concentration when compared to the Aβ_25−35_ group. In the Aβ_25−35_ + limonin group at doses of 12.5, 25, 50 μg/mL, the concentration of Ca^2+^ declined from 25.3% to 6.55%, indicating that limonin could antagonize the release of Ca^2+^ in Aβ_25−35_-treated PC12 cells. Additionally, there was a dose-dependent relationship between the decline in the concentration of intracellular Ca^2+^ and the concentration of limonin utilized for the treatment of cells (*p* < 0.0001).

### ROS Content in PC12 Cells

Figure 4B showed that the level of ROS significantly increased (*p* < 0.0001) in PC12 cells that were exposed to Aβ_25−35_, compared to the control group cells. Pre-treatment with limonin for 24 h prior to the addition of 20 μM of Aβ_25−35_ to the cells resulted in decreased content of ROS (*p* < 0.0001). In the Aβ_25−35_ + limonin group at a dose of 12.5 to 50 μg/mL, the fluorescence intensity of ROS decreased from 2263.67 to 780.00 with increasing concentration of limonin, indicating that limonin can significantly reduce ROS level in Aβ_25−35_-treated PC12 cells. When the concentration of limonin was at 50 μg/mL, compared to the Aβ_25−35_ group, the fluorescence intensity of ROS showed a significant decrease (*p* < 0.0001).

### MMP in PC12 Cells

The change of MMP in PC12 cells was measured by the JC-1 staining assay in Figure 4C. Compared to the control group, MMP significantly decreased after treatment with 20 μM of Aβ_25−35_ (*P* < 0.0001), which was confirmed by the increase of red JC-1 staining in the cells of the Q3 area. The pre-treatment of limonin significantly reversed the decrease of MMP caused by Aβ_25−35_ treatment (*P* < 0.0001). In the Aβ_25−35_ + limonin group at low, medium and high doses, the change of MMP was dose-dependent with the concentration of limonin.

### Apoptosis of PC12 Cells

To elucidate whether Aβ_25−35_ induced death of PC12 cells is via an apoptotic-like mechanism, Annexin V-FITC/PI double staining method combined with flow cytometry technology was used to analyze the apoptosis of cells. As shown in Figure 4D, the total apoptotic rate of the control group was 4.85%, while 20 μM Aβ_25−35_ significantly increased the early and late apoptosis of PC12 cells, and the total apoptotic rate reached 42.5% (*P* < 0.0001). After pre-protecting PC12 cells with different concentrations of limonin (12.5, 25, and 50 μg/mL), the total apoptotic rate of PC12 cells decreased to 31.7, 20.63, and 9.66%, respectively, compared with the Aβ_25−35_ group, indicating that limonin could significantly inhibit Aβ_25−3_-induced apoptosis of PC12 cells in a concentration-dependent manner.

### The Activation of Bax/Bcl-2 and Caspase-3 in PC12 Cells

**Figure 5:**
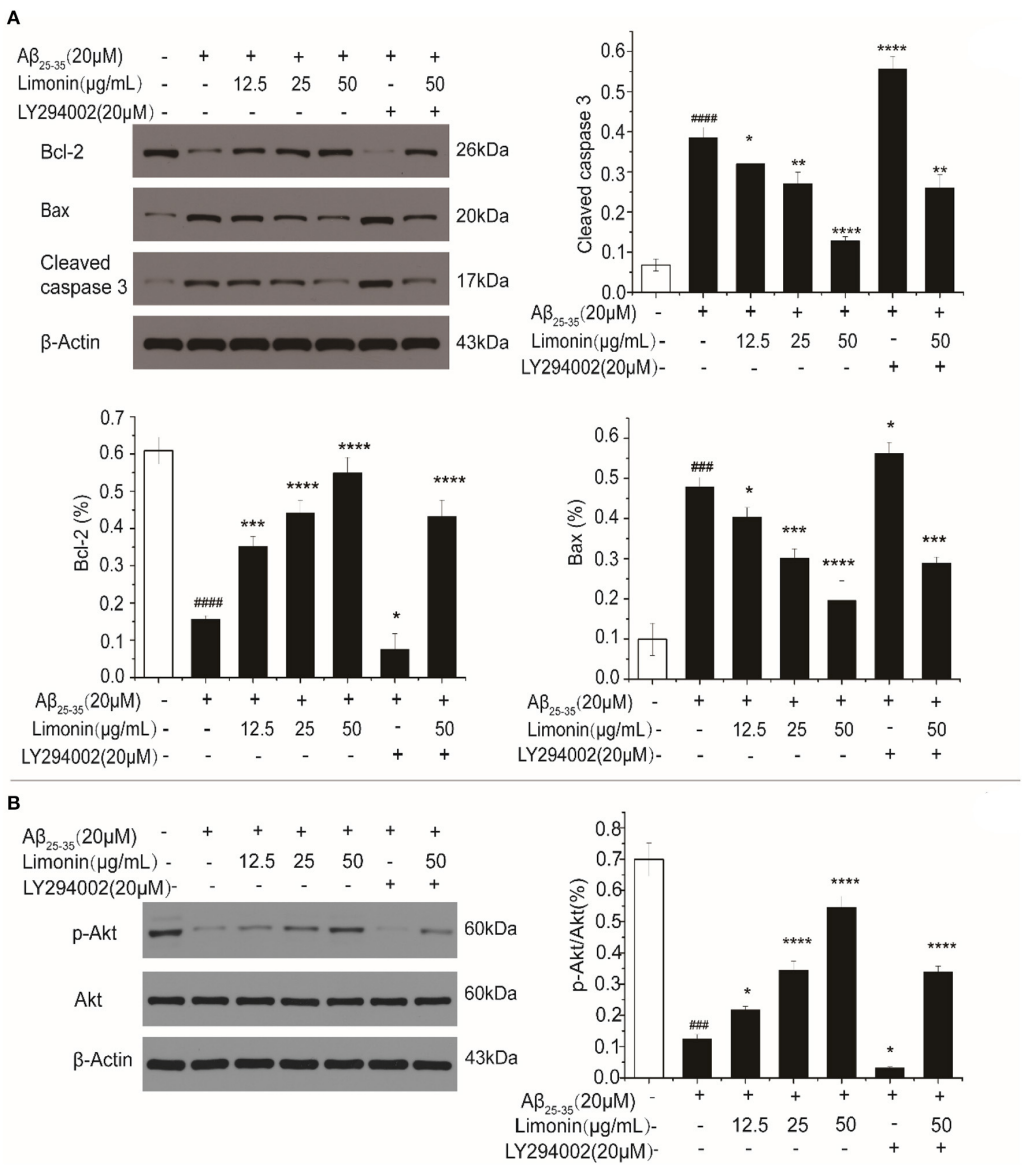
The expression levels of Bax/Bcl-2 and Caspase-3 **(A)**, as well as p-Akt **(B)** in PC12 cells determined by Western blot. The values represent the mean ± SD (*n* = 3 in each group). ###*p* < 0.001 and ####*p* < 0.0001 compared with the control group; *p < 0.05, **p < 0.01, ***p < 0.001 and ****p < 0.0001 compared with the Aβ_25−35_ group.

Bcl-2 and Caspase family proteins are related to cell apoptosis. In the current study, Western blot was used to determine Bax, Bcl-2 and Caspase-3 proteins under different treatment conditions. As shown in Figure 5A, when compared to control cells, PC12 cells that were treated with Aβ_25−35_ for 48 h showed a significant decrease in the expression of Bcl-2, a significant increase in the expression of Bax and an increase in the ratio of Bax/Bcl-2 (*P* < 0.0001). Meanwhile, cleaved caspase-3 was also up-regulated (*P* < 0.0001), indicating that PC12 cells were undergoing apoptosis. In the limonin pre-treatment group, the expression of Bax was down-regulated, Bcl-2 was up-regulated, and cleaved caspase-3 was down-regulated, indicating the antagonistic effect of limonin on Aβ_25−35_-mediated cell growth inhibition. As compared to the LY294002 inhibitor group, limonin was found to antagonize the inhibitor's regulation of apoptotic proteins, thereby preventing the occurrence of apoptosis programs.

### PI3K/Akt Signal Pathway Activation in PC12 Cells

In order to explore the potential intracellular signaling mechanism of limonin antagonizing Aβ_25−35_-induced death of PC12 cells, the changes in PI3K/Akt activation were evaluated. As shown in Figure 5B, compared to the control cells, the levels of p-Akt protein in Aβ_25−35_ -treated PC12 cells were down-regulated and p-Akt/Akt ratio was significantly decreased (*P* < 0.0001). On the contrary, the expression level of p-Akt protein and the ratio of p-Akt/Akt in limonin treated PC12 cells were increased, indicating that limonin promoted the activation of Akt to form p-Akt, which then stimulated the biological activity of the PI3K/Akt signaling pathway in PC12 cells. LY294002 is a specific inhibitor of this pathway. LY294002 was added 1 h before limonin pre-treatment. It was found that the inhibitor LY294002 reversed the upregulation of p-Akt by limonin, indicating that limonin plays a role in activating the PI3K pathway.

## Discussion

In order to improve the utilization rate of pomelo seed, more high value-added products have to be found and applied. Firstly, the identification of chemical components of pomelo seeds extracts was investigated using UPLC-MS/MS. This study found that pomelo seeds are rich in active substances, such as limonoids, flavonoids, phenolic compounds, coumarin derivatives, among others. The main ingredient limonin was successfully obtained with a purity of 99.13% via using crystallization and preparative-HPLC technologies. This high purity sample was helpful to understand the action mechanism of limonin directly and ensure the correctness of results.

In previous studies, limonin was shown to be able to improve the learning and memory ability of naturally aging rats, increase the anti-oxidant capacity of brain tissue, and have an effect of delaying brain aging (25, 26). The main clinical manifestations of AD are slowness of action and memory decline (27), thus the study of limonin is of great significance for exploring the prevention and dietary health care of AD. In the current study, an *in vitro* experiment was conducted to explore the role of limonin in antagonizing the neurotoxicity of PC12 cells mediated by Aβ_25−35_. The experimental data showed that Aβ_25−35_ significantly inhibited cell viability and increased lactate release. LDH represents the structural integrity of cell membrane to a certain extent (28). The increase in the content of LDH in the culture medium indicated that the cell membrane was damaged. Pre-treating cells with limonin could inhibit the neurotoxicity caused by treatment with Aβ_25−35_. These results showed that limonin could protect PC12 cells from Aβ_25−35_-mediated cytotoxicity and damage.

Precipitation of Aβ could increase the level of Ca^2+^ in the brain neurons of AD patients, reduce the excitability threshold of neurons, and cause neuronal function damage (29). We observed the protective effect of limonin pre-treatment by a significant decrease of Ca^2+^, compared with Aβ_25−35_ treatment group. ROS was closely related to AD pathological Aβ deposition, Tau protein phosphorylation, and inflammation (30). Mitochondria are the main source to generate ROS, thus mitochondrial dysfunction could easily lead to production and accumulation of ROS and cause cell oxidative damage (31). In PC12 cells treated with Aβ_25−35_, a significant increase in ROS and a decrease in MMP were observed, indicating a mitochondrial system dysfunction. On the contrary, limonin treatment significantly reduced the excessive production of ROS, reversed the trend of MMP decline, and ultimately protected PC12 from Aβ_25−35_-mediated oxidative stress and damage. This study has shown that limonin participated in the elimination of ROS, thereby inhibiting the occurrence of peroxidation and maintaining the integrity of cell membranes.

The neurotoxicity caused by abnormal deposition of Aβ could cause apoptosis of nerve cells, which is the main cause of AD (32). In PC12 cells treated with Aβ_25−35_, the apoptotic rate was found to increase significantly, while the apoptotic rate significantly decreased after limonin treatment, indicating that limonin could reverse the occurrence of cell apoptosis. In the past, studies on cell apoptosis mainly focused on DNA degradation by enzymes into oligonucleotide fragments during cell apoptosis (33). In recent years, there has been more interest in investigating the early processes of cell apoptosis. Caspase participates in the process of specific enzymolysis and mediates the apoptosis signal transduction pathway. The roles of Caspase-3 and Bcl-2 have attracted much attention. Bcl-2 and Bax are a pair of homologous genes with opposite functions. In Aβ_25−35_ treatment group, Bcl-2 expression was down regulated; Bax and Caspase-3 protein expression was up-regulated. It is generally believed that the role of Bcl-2 is upstream of the activation of Caspase-3. Caspase-3 is a protease for enzymatic hydrolysis of downstream substrates of each apoptotic pathway. Therefore, inhibiting the activation of Caspase-3 can lead to suppression of cell apoptosis. When the expression of Bcl-2 decreased, the cells will undergo apoptosis. The results of the limonin pre-treatment group showed that the changes of the above-mentioned proteins caused by Aβ_25−35_ were reversed, and apoptosis was thereby prevented by limonin. It could be speculated that limonin inhibited the cascade of Bcl-2 and Caspase family proteins, and ultimately prevented cell apoptosis. This conclusion can also be verified by the results with LY294002.

PI3K/Akt is an important signal transduction pathway for anti-apoptosis and plays an important role in promoting cell survival. The protective effect of some drugs was exerted by regulating the Bcl-2 signal pathway and affecting the phosphorylation of Akt (34, 35). A large amount of literature showed that the PI3K/Akt pathway is closely related to neurological diseases such as AD and Parkinson disease. Qi et al. reported that schisandrin and nootkatone showed a neuroprotective effect via PI3K/Akt pathway, inhibiting inflammation and apoptosis (36). Lou et al. reported that linarin prevented Aβ_25−35_ from inducing neurotoxicity via PI3K/Akt pathway (37). Akt is a serine/threonine protein kinase that promotes cell survival after nerve injury through PI3K signal transduction pathways. Akt activates to form p-Akt, which can phosphorylate multiple downstream targets (38). Therefore, the PI3K/Akt signaling pathway plays an anti-apoptotic effect through the apoptosis pathway and affects downstream anti-apoptotic molecules. In this study, after treatment with Aβ_25−35_, the expression of p-Akt was significantly down-regulated, and the ratio of p-Akt/Akt decreased. With pre-treatment by limonin, the expression of p-Akt and the ratio of p-Akt/Akt were significantly increased. However, when the PI3K/Akt inhibitor LY294002 was added, the protective effect of limonin was attenuated, indicating that limonin exerted neuroprotective effects by affecting the PI3K/Akt pathway.

## Conclusions

In conclusion, to begin with, the phenols compositions of waste pomelo seed extract were analyzed using UPLC-MS/MS. Besides, limonin with high concentration in pomelo seed was successfully obtained with a purity of 99.13% via using crystallization and preparative-HPLC technologies. Finally, limonin can protect nerve cells by activating the PI3K/Akt pathway. This activation also prevented mitochondrial damage, protected nerve cells from oxidative damage, and effectively reduced Aβ-induced neurotoxicity. This information provides a practical insight to produce valuable products from discarded pomelo seed, possessing potential to increase the economic profit and resource utilization rate while reducing environmental waste.

## Data Availability Statement

The original contributions presented in the study are included in the article/Supplementary Material, further inquiries can be directed to the corresponding author/s.

## Author Contributions

YQ: data curation, formal analysis, investigation, roles/writing—original draft, validation, project administration, supervision, and resources. JY: data curation, formal analysis, investigation, and roles/writing—original draft. LM: data curation, formal analysis, investigation, writing—review and editing, and validation. MS: formal analysis, writing—review and editing, and validation. GL: data curation, formal analysis, writing—reviewing and editing, investigation, supervision, and project administration. All authors contributed to the article and approved the submitted version.

## Funding

This study was supported by the Guangdong Provincial Key Laboratory of Lingnan Specialty Food Science and Technology (2021B1212040013), and the Guangdong Province Key Field R&D Program Project (2019B020212002). We thank Zhongkai University of Agricultural Engineering for providing experimental conditions.

## Conflict of Interest

The authors declare that the research was conducted in the absence of any commercial or financial relationships that could be construed as a potential conflict of interest.

## Publisher's Note

All claims expressed in this article are solely those of the authors and do not necessarily represent those of their affiliated organizations, or those of the publisher, the editors and the reviewers. Any product that may be evaluated in this article, or claim that may be made by its manufacturer, is not guaranteed or endorsed by the publisher.
