# Supplementary figures and images for: Limonin Isolated From Pomelo Seed Antagonizes Aβ25-35-Mediated Neuron Injury via PI3K/AKT Signaling Pathway by Regulating Cell Apoptosis
Source: Front Nutr. 2022 May 12;9:879028. doi: 10.3389/fnut.2022.879028 (PMC9133815; doi:10.3389/fnut.2022.879028)

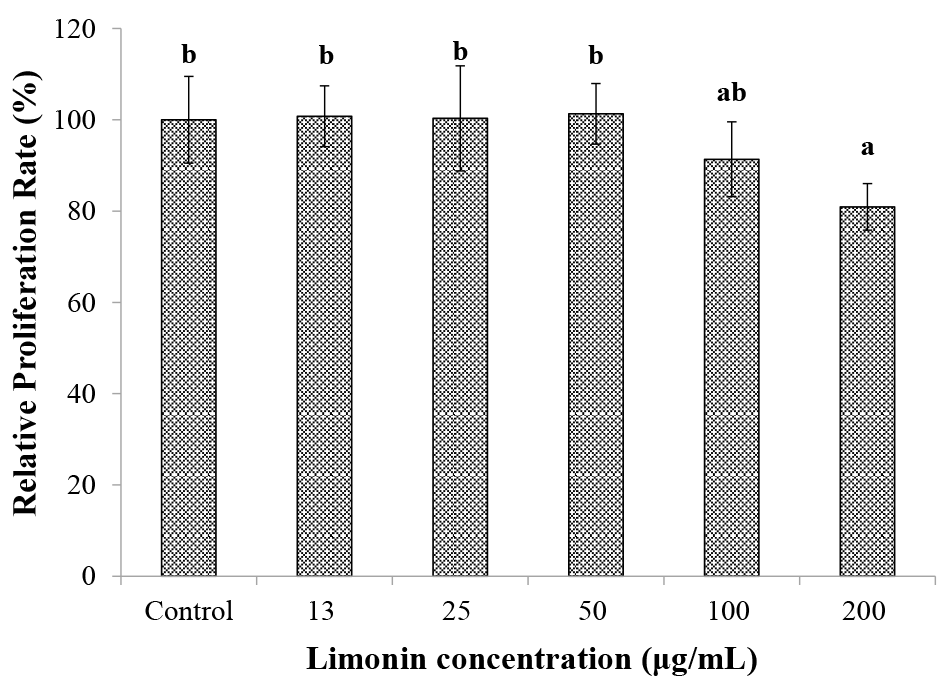

Supplement: Supplementary file 5 [file Image_1.TIF]

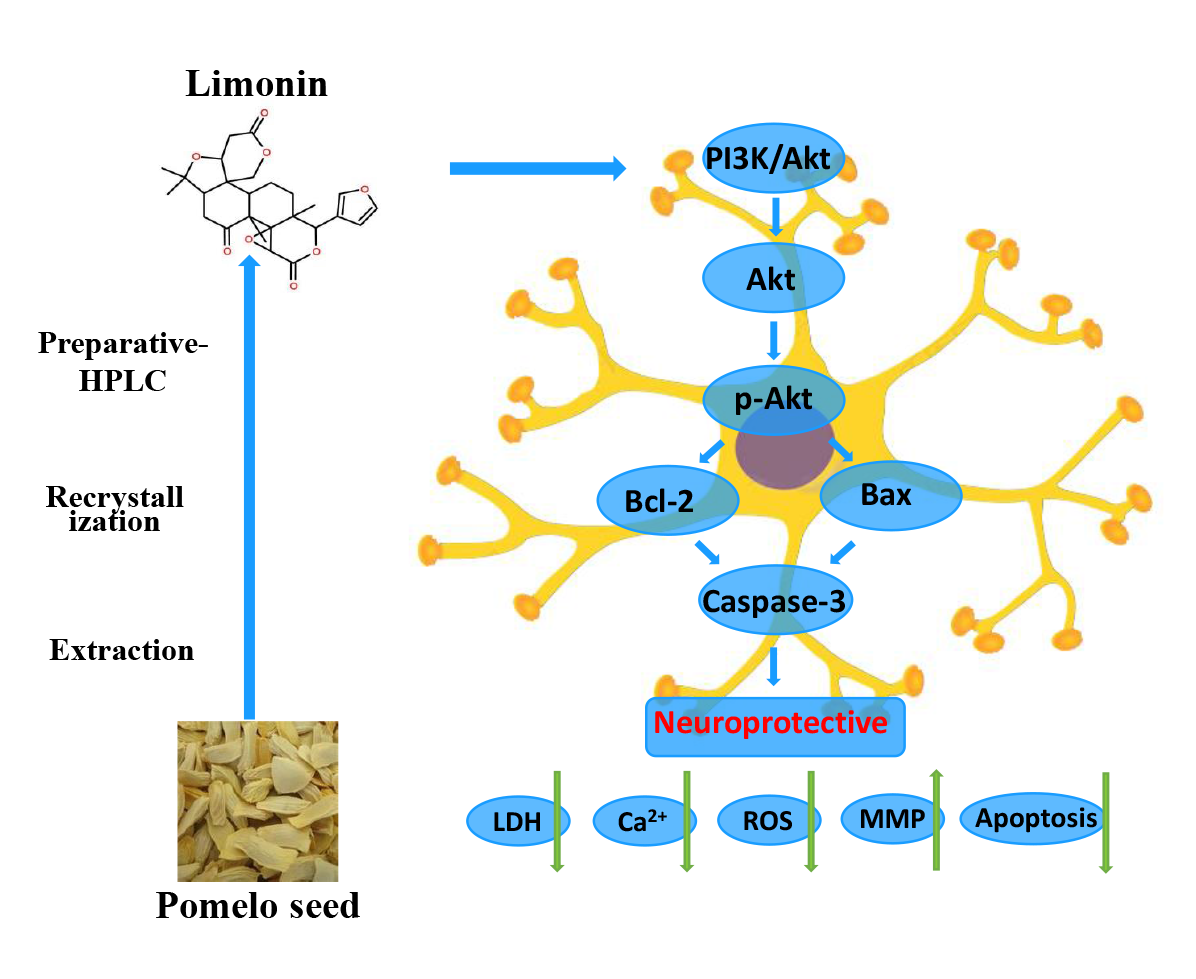

Supplement: Supplementary file 6 [file Image_2.TIF]
